# Chemical Composition and Cytotoxicity Evaluation of Essential Oil from Leaves of *Casearia Sylvestris*, Its Main Compound α-Zingiberene and Derivatives

**DOI:** 10.3390/molecules18089477

**Published:** 2013-08-08

**Authors:** Diego Dinis Bou, João Henrique G. Lago, Carlos R. Figueiredo, Alisson L. Matsuo, Rafael C. Guadagnin, Marisi G. Soares, Patricia Sartorelli

**Affiliations:** 1 Instituto de Ciências Ambientais, Químicas e Farmacêuticas, Universidade Federal de São Paulo, Diadema 09972-270, SP, Brazil; 2 Disciplina de Biologia Celular, Departamento de Micro, Imuno e Parasitologia, Universidade Federal de São Paulo, São Paulo 04021-001, SP, Brazil; 3 Instituto de Química, Universidade Federal de Alfenas, Alfenas 37130-000, MG, Brazil

**Keywords:** *Casearia sylvestris*, essential oil, α-zingiberene, zingiberene derivatives, cytotoxic activity

## Abstract

*Casearia sylvestris* (Salicaceae), popularly known as “guaçatonga”, is a plant widely used in folk medicine to treat various diseases, including cancer. The present work deals with the chemical composition as well as the cytotoxic evaluation of its essential oil, its main constituent and derivatives. Thus, the crude essential oil from leaves of *C. sylvestris* was obtained using a Clevenger type apparatus and analyzed by GC/MS. This analysis afforded the identification of 23 substances, 13 of which corresponded to 98.73% of the total oil composition, with sesquiterpene α-zingiberene accounting for 50% of the oil. The essential oil was evaluated for cytotoxic activity against several tumor cell lines, giving IC_50_ values ranging from 12 to 153 μg/mL. Pure α-zingiberene, isolated from essential oil, was also evaluated against the tumor cell lines showing activity for HeLa, U-87, Siha and HL60 cell lines, but with IC_50_ values higher than those determined for the crude essential oil. Aiming to evaluate the effect of the double bonds of α-zingiberene on the cytotoxic activity, partially hydrogenated α-zingiberene (PHZ) and fully hydrogenated α-zingiberene (THZ) derivatives were obtained. For the partially hydrogenated derivative only cytotoxic activity to the B16F10-Nex2 cell line (IC_50_ 65μg/mL) was detected, while totally hydrogenated derivative showed cytotoxic activity for almost all cell lines, with B16F10-Nex2 and MCF-7 as exceptions and with IC_50_ values ranging from 34 to 65 μg/mL. These results indicate that cytotoxic activity is related with the state of oxidation of compound.

## 1. Introduction

The genus *Casearia* was traditionally regarded as belonging to the family Flacourtiaceae. However phylogenetic and chemical studies, besides ecological and morphological observations showed that Flacoutiaceae is, in fact, composed by two clades, with the genus *Casearia* belonging to the Salicaceae [1,2]. This genus is found especially in tropical and subtropical regions, like most other native species found in Brazil. This genus has more than 160 described species of which 70 belong to the American continent and 37 are present in Brazil [3].

*Casearia sylvestris* Sw. is popularly known as “guaçatonga” and is utilized in traditional medicine to treat several pathologic processes such as inflammation, skin lesions and microbial infections. The leaves and bark are considered a purgative tonic, and anti-rheumatic [4]. This species also showed anti-inflammatory, antimicrobial, antiplasmodial and anti-ulcer properties detected by *in vitro* and pre-clinical studies [5]. Currently, this medicinal plant is included in the arsenal of “folk herbal medicines”.

Phytochemically the genus *Casearia* has been characterized by the occurrence of biologically active metabolites. From *C. clarkei* podophyllotoxin-type lignans [6], a class of compounds which displayed anti-tumor activity and important antineoplasic properties [7,8] has been described. Coumarins have been described in *C. graveolens* [9] and they showed biological activities, mainly antitumoral and anti-HIV [10]. Diterpenes, especially clerodane derivatives, has also been described in species of *Casearia* [11,12,13]. *C. sylvestris* accumulates mainly clerodane diterpene derivatives named casearins and casearvestrins [14,15,16,17,18,19,20], which have been described to have cytotoxic activity against V-79 cells, being casearin C the most active, and one whose structure was characterized by the lack of an oxygen-bearing function at C-6 [14,15]. Casearvestrins A-C also showed cytotoxic activity against KB tumor cells (human oral epidermoid carcinoma), being casearvestrin C the most active [16].

The essential oil of *C. sylvestris* also has had its composition and chemical variability analyzed, and the predominance of sesquiterpenes was described, being germacrene D [21] or bicyclogermacrene and β-caryophyllene [22] the main compounds. Additionally, this oil showed a good selective cytotoxicity against HeLa, A-549 and HT-29 tumor cells, while the oil causes hemolysis in several kinds of erythrocytes [22]. As part of our ongoing studies of volatile oils from Brazilian species [23,24,25,26], the present work deals with the chemical composition of the essential oil from *C. sylvestris* as well as the cytotoxic evaluation of the crude oil, its main component α-zingiberene and its hydrogenated derivatives against several tumor cell lines.

## 2. Results and Discussion

Drug discovery from plants still provides important new drugs, especially for cancer. Using this approach, several plants with metabolites that exhibit antitumor activity have been described [27]. Aiming at the discovery of new anti-tumoral compounds from Brazilian flora, fresh leaves of *C. sylvestris* were extracted by steam distillation for 5 h, giving 2.92 g of a pale yellow oil (corresponding to a yield of 0.23% w/w). The analysis of its chemical components was performed by gas chromatography associated with mass spectrometry using a mass spectra library (LIB NIST107) for identification and comparison with the mass spectra available in the literature [28], strictly considering fragmentation patterns, and Kovats indexes of each constituent. This analysis allowed the identification of 23 substances (Table 1), corresponding to 98.73% of the total oil composition. The oil was shown to be composed mainly of α-zingiberene (48.31%) followed of the *E*-caryophyllene (14.27%), γ-muurolene (5.16%), viridiflorene (5.07%), and acoradiene (4.11%), contrary to previously analyzed essential oil, in which bicyclogermacrene, with about 41%, followed by β-caryophyllene (18%) and spathulenol (16%) were described as the major components [22,29]. Other constituents that were also observed as major components of the essential oil of *C. sylvestris* were β-elemene (31.7%) [30] and germacrene D [21]. These differences in chemical composition can be attributed, at least in part, to several factors such as the presence of certain micronutrients in the soil, temperature, environmental factors and chemotypes [31].

**Table 1:** Chemical composition of the essential oil from leaves of *C. sylvestris*.

Compound	KI	% Composition
δ-elemene	1339	0.29
α-copaene	1376	0.20
β-elemene	1391	2.19
α-*cis*-bergamotene	1415	0.24
*E*-caryophyllene	1418	14.27
gurjunene	1432	0.16
α-guaiene	1439	1.50
α-humulene	1454	1.68
α-patchoulene	1456	1.08
α-acoradiene	1463	4.11
β-acoradiene	1466	2.30
γ-muurolene	1477	5.16
viridiflorene	1493	5.07
α-zingiberene	1495	48.31
β-*trans*- guaiene	1500	1.33
7-*epi*-α-selinene	1517	1.05
δ-cadinene	1524	1.33
germacrene B	1556	0.39
khusimone	1593	0.25
10-*epi*-γ-eudesmol	1619	0.35
1-*epi*-cubenol	1627	0.51
α-muurolol	1645	3.72
α-cadinol	1653	3.24
TOTAL		98.73

Considering that α-zingiberene represents about 50% of crude oil from *C*. *sylvestris*, and in order to evaluate the biological activities of the pure component, this material was fractionated over a SiO_2_/AgNO_3_ column to afford 200 mg of α-zingiberene, which was identified by ^1^H-, ^13^C-NMR and LREIMS. 

**Figure 1:**
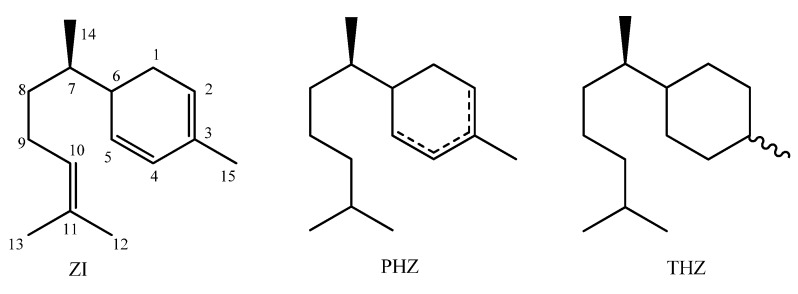
Structures of α-zingiberene (ZI), partially hydrogenated zingiberene (PHZ) and totally hydrogenated **z**ingiberene (THZ).

In the ^1^H-NMR spectrum of α-zingiberene signals assigned to the olefinic protons were observed at δ 5.38 (*br s*, H-2), δ 5.57 (*dd*, *J* = 9.8 and 2.6 Hz, H-4), δ 6.70 (*dd*, *J* = 9.8 and 2.0 Hz, H-5) and δ 5.02 (*br t*, *J* = 7.0 Hz, H-10). Hydrogens of the methyl groups linked to the sp^2^ carbons of double bonds C-12, C-13 and C-15 showed peaks at δ 1.61 (*s*), δ 1.64 (*s*) and δ 1.68 (*d*, *J* = 2.0 Hz), respectively, while methyl hydrogens of the C-14 was detected at δ 0.83 (*d*, *J* = 6.2 Hz). Additional peaks ranging from δ 1.16 to δ 2.14 correspond to remaining hydrogens (H-1, H-6, H-7, H-8 and H-9). The ^13^C-NMR spectrum showed fifteen signals, being six at the region of double bonds (δ 120–135), referring to carbons C-2, C-3, C-4 C-5, C10 and C-11, besides signals at δ 26.2, 17.9, 17.8 and 21.3 corresponding to methyl groups in C-12, C-13, C14 and C-15 respectively, thus confirming its structure as α-zingiberene (Figure 1) [32,33].

α-Zingiberene was further subjected to analysis by LREIMS and its mass spectrum showed the molecular ion [M^+^] at *m/z* 204, confirming the formula C_15_H_24_, which characterizes the compound as α-zingiberene [28]. The initial fragmentation with the loss of hexenyl radical (C_6_H_11_) with *m/z* 83 generates the fragment at *m/z* 121. The additional loss of a molecule of H_2_ with simultaneous rearrangement generates the observed base peak at *m/z* 119. The peak at *m/z* 91 indicates the formation of a benzene ring with alkyl side chain, the cation tropilium. Despite the occurrence of α-zingiberene as the most important compound of the oil of ginger (*Zingiber officinale*) [32], this is the first reported occurrence of this sesquiterpene as the main constituent in the essential oil from leaves of *C. sylvestris*.

**Scheme 1:**
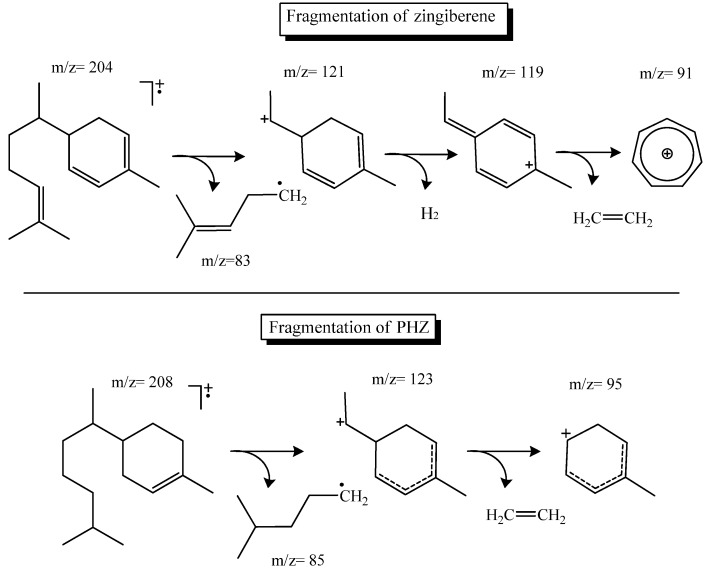
Fragmentation of LREIMS spectra of α-zingiberene and PHZ.

Aiming at establishing the relationships between the structure/activity of α-zingiberene, this was subjected to a double bond reduction reaction by catalytic hydrogenation. Initially α-zingiberene was mixed with H_2_ and Raney-Ni catalyst. After purification the product was subjected LREIMS analysis to confirm the hydrogenation. The mass spectrum revealed a molecular ion at *m/z* 208, *i.e.*, four units of mass higher than the α-zingiberene, indicating that only two double bonds were hydrogenated. The mass spectrum indicated the loss of a hexyl fragment radical (*m/z* 85) to give a monounsaturated [C_9_H_15_]^+^ cation peak at *m/z* 123. A further fragmentation of this peak with loss of ethylene produced the base peak of spectrum *m/z* 95 (Scheme 1). This pattern indicated the reduction of the exocyclic double bond and one of the ring alkenes, suggesting that the partially hydrogenated product corresponds to the PHZ structure shown in Figure 1. α-zingiberene was also further subjected to hydrogenation under more drastic conditions. LREIMS analysis of the purified product showed the molecular ion at *m/z* 210, confirming that α-zingiberene was fully hydrogenated, as shown in Figure 1 (THZ).

The crude oil as well as purified α-zingiberene were evaluated for its antitumor activity *in vitro*, initially against murine melanoma cell line B16F10-Nex2. The obtained results indicated that the essential oil was cytotoxic against this cell line with an IC_50_ value of 61.5 μg/mL. Further, the cytotoxic potential of the crude oil, α-zingiberene and their hydrogenated derivatives were evaluated against different human tumor cell lines (Table 2). Positive control assay was carried out with cisplatin (standard drug), with IC_50_ of 176.0 μg/mL on B16F10-Nex2 cells.

**Table 2:** IC_50_ (µg/mL) values to essential oil from leaves of *C. sylvestris*, zingiberene and derivatives (partially hydrogenated zingiberene-PHZ and totally hydrogenated zingiberene-THZ) as well as positive control (cisplatin) against cell lines.

Sample	A2058	HeLa	MCF-7	U-87	Siha	HL60	B16F10 Nex2
Crude oil	41.1 ± 2.0	29.0 ± 0.0	42.2 ± 3.4	27.1 ± 1.5	23.9 ± 0.46	12.0 ± 1.9	61.5 ± 1.8
ZI	>200	63.2 ± 2.7	>200	153.0 ± 29.9	48.0 ± 0.8	98.7 ± 9.7	27.0 ± 1.2
PHZ	>200	>200	>200	>200	160	>200	65.2 ± 1.1
THZ	64.1 ± 3.0	65.6 ± 1.4	131.3 ± 10.0	59.4 ± 3.5	54.9 ± 2.8	34.3 ± 0.9	>200
Cisplatin (control)	nd	20.3 ± 1.20	nd	44.9 ± 6.0	59.8 ± 0.0	20.9 ± 1.50	52.6 ± 4.49

ZI – purified zingiberene, PHZ – partially hydrogenated zingiberene, THZ – totally hydrogenated zingiberene, nd – not determined.

The crude oil displayed activity against all cell lines tested with IC_50_values ranging from 12 to 42 μg/mL, being HL60 the most sensitive cell line to the crude oil, with an IC_50_ of 12.0 μg/mL. Otherwise, the purified α-zingiberene from the essential oil showed a cytotoxic activity against HeLa, U-87, Siha and HL60 cell lines, but the IC_50_ values were higher than those obtained for the crude oil, indicating that there may be a synergism or additive activity between α-zingiberene and other substances present in the crude oil for the observed activity against these cell lines. However, when evaluated against B16F10-Nex2 cell line, α-zingiberene cytotoxicity was intensified, with an IC_50_ value of 27.0 μg/mL, compared to the crude oil (61.5 μg/mL), suggesting that this sesquiterpene may be primarily responsible for the cytotoxic activity against this particular cell line.

Additionally, it was observed that at the tested concentrations PHZ was only active against the B16F10-Nex2 cell line, with an IC_50_ of 65.2 μg/mL, and IC_50_ values greater than 200 μg/mL for the other tumor cell lines, indicating that PHZ has low cytotoxic activity against these tumor cells.

The totally hydrogenated zingiberene (THZ) showed cytotoxic activity against almost cell lines, except for B16F10-Nex2 and MCF-7, with IC_50_ values ranging from 34–65 μg/mL. The higher lipophilicity of totally hydrogenated zingiberene, compared to other compounds, could be responsible for enhanced cell penetration through the plasma membrane. 

Although there are no studies concernng the cytotoxic activity of sesquiterpenes isolated from oil of *C. sylvestris*, Salvador *et al*. [34] showed that essential oil from *C. lasiophylla* were cytotoxic against tumor cell lines U251, UACC-62, MCF-7, NC1-ADR/RES, NCI-H460, PC03, OVCAR-3, HT-29 K562. Silva *et al*. [22], also showed the cytotoxic activity of the crude essential oil of leaves of *C. sylvestris* collected in Campinas against several tumor cell lines (A-549, HT-29 and HeLa). However, these oils were shown to be composed by bicyclogermacrene and β-caryophyllene as main compounds, a different profile of that detected in the present work.

## 3. Experimental

### 3.1. Plant Material

Leaves of *Casearia sylvestris* with no deformation and degradation by exogenous factors were selected and collected with the aid of sterile pliers from a single tree at the Atlantic Forest area in São Paulo city, SP, Brazil (coordinates 23 53'08.86''S, 46 40'10.45''O), in October, 2012.

### 3.2. Essential Oil Extraction and Analysis

Fresh leaves (1,278 g) of *C. sylvestris* were extracted over 5 h by steam distillation in a Clevenger type apparatus to afford 2.9 g of crude essential oil. The oil was analyzed by GC-FID and GC-LR-EI-MS using a RtX-5 capillary column. The identification of the individual compounds was performed by comparison of retention indexes (determined relatively to the retention times of a series of *n*-alkanes) and comparison of recorded mass spectra with those available in the system library [28]. GC chromatograms were obtained on a Shimadzu GC-2010 gas chromatograph equipped with an FID-detector and an automatic injector (Shimadzu AOC-20i) using a RtX-5 (5% phenyl, 95% polydimethylsiloxane (Restek, Bellefonte, PA, USA, 30 m × 0.32 mm × 0.25 μm film thickness) capillary column. These analyses were performed by injecting 1.0 μL of a 1.0 mg/mL solution of volatile oil in CH_2_Cl_2_ in a split mode (1:10) employing helium as the carrier gas (1 mL/min) under the following conditions: injector and detector temperatures of 220 °C and 250 °C, respectively; oven programmed temperature from 40–240 °C at 3 °C/min, holding 5 min at 240 °C. GC-FID was performed in quantitative analysis. The percentage compositions of the oil samples were computed by internal normalization from the GC peak areas without using correction for response factors. GC-LR-EI-MS analysis was conducted on a Shimadzu GC-17A chromatograph interfaced with a MS-QP-5050A mass spectrometer. Helium was used as the carrier gas. The LR-EI-MS operating conditions were an ionization voltage of 70 eV and an ion source temperature of 230 °C with the same conditions described above.

### 3.3. Chromatographic Separation Procedures

Part of crude oil (2.0 g) was subjected to flash chromatography on SiO_2_ gel soaked with AgNO_3_ (10%) column (63 cm × 5 cm i.d.) chromatography eluted with CH_2_Cl_2_-acetone in proportions of 99:1, 95:5 and 90:10 (200 mL for each eluent) to afford 30 fractions which were individually analyzed using GC-FID and then pooled into nine groups (A to I). Fraction F was composed of pure α-zingiberene (203 mg).

*α-Zingiberene*. ^1^H-NMR (δ, CDCl_3_, Bruker DPX300, 300 MHz): 2.01 *m* (2H, H-1), 5.38 *brs* (1H, H-2), 5.57 *dd* (*J* = 9.8; 2.6 Hz, 1H, H-4), 6.70 *dd* (*J* = 9.8; 2.0 Hz, 1H, H-5), 2.14 *m* (1H, H-6), 1.16 *m* (1H, H-7), 1.48 *m* (2H, H-8), 1.99 *m* (2H, H-9), 5.02 *brt* (*J* = 7.0 Hz, 1H, H-10), 1.61 *s* (3H, H-12), 1.64 *s* (3H, H-13), 0.83 *d* (*J* = 6.2 Hz, 3H, H-14), 1.68 *d* (*J* = 2.0 Hz, 3H, H-15). ^13^C-NMR (δ, CDCl_3_, 75 MHz): 26.2 (C-1), 120.6 (C-2), 131.6 (C-3), 128.1 (C-4), 131.3 (C-5), 38.3 (C-6), 34.5 (C-7), 36.3 (C-8), 24.7 (C-9), 125.0 (C-10), 131.4 (C-11), 26.2 (C-12), 17.9 (C-13), 17.8 (C-14), 21.3 (C-15). LR-EI-MS (70 eV): *m/z* (rel. int.): 204 (9), 145 (26), 132 (90), 119 (98), 105 (62), 91 (43), 55 (47).

### 3.4. Hydrogenation of α-zingiberene

**Figure 2:**
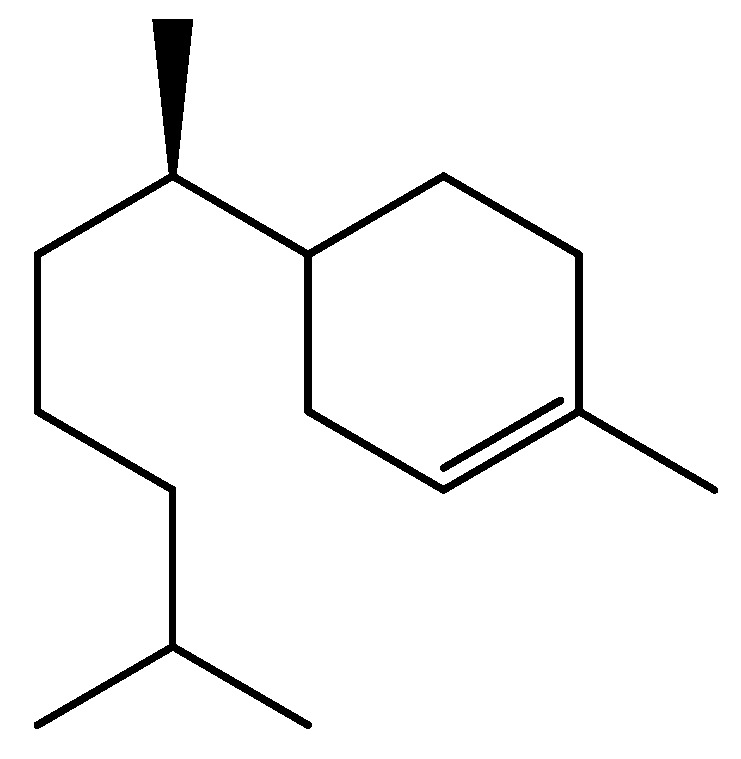
Tentative structure of PHZ.

In a high-pressure reactor (stainless steel), pure α-zingiberene (90 mg, 0.44 mmol), Ni-Raney catalyst (10%, 9 mg; Aldrich; pore size: 50 μm, superficial area: 80–100 m^2^/g) and hexane (5 mL) were added. After addition of H_2_ (8 atm), the mixture were stirred for 3 h at 90 °C. Then, the reactor was cooled and the product was filtered [35]. After solvent evaporation, 30 mg (33%) of a colorless oil was obtained, which was characterized as PHZ. ^1^H-NMR (δ, CDCl_3_): 1.60 m (2H, H-1), 2.01 m (2H, H-2), 5.32 m (1H, H-4), 2.04 m (2H, H-5), 1.63 m (2H, H-6), 1.16 m (1H, H-7), 1.25 m (4H, H-8, H-9), 1.23 m (2H, H-10), 1.62 m (1H, H-11), 0.91 d (*J* = 6.2 Hz; 6H, H-12 and H-13), 0.86 d (*J* = 6.5 Hz, 3H, H-14), 1.66 s (3H, H-15). This data suggests that the remaining double bond is located as shown in Figure 2, but there are some inconsistencies of these multiplicities with the theoretical spectrum and we did not have sufficient sample for more extensive NMR studies that would have allowed us to confirm this structure unambiguously. This product was fully hydrogenated, under more drastic conditions (26 atm H_2_, Ni-Raney 20%, 30 mg, 120 °C, 3 h) to obtain 60 mg (65%) of the fully saturated product THZ. 

### 3.5. Cell Lines

The murine melanoma cell line B16F10 was originally obtained from the Ludwig Institute for Cancer Research (São Paulo, Brazil). The melanotic B16F10-Nex2 subline, characterized at the Experimental Oncology Unit (UNIFESP-Federal University of São Paulo), is characterized by low immunogenicity and moderate virulence. Human melanoma (A2058), glioblastome (U87), leukemia (HL-60), uterus carcinoma (Siha) and breast cancer (MCF-7) cell lines were obtained from the Ludwig Institute for Cancer Research. Human cervical carcinoma (HeLa) was acquired from Dr. Hugo Pequeno Monteiro (UNIFESP).

### 3.6. *In Vitro* Cytotoxic Activity

The essential oils extracted from leaves of *C. sylvestris*, as well as pure α-zingiberene, zingiberene partially hydrogenated and zingiberane, were resuspended in dimethyl sulfoxide (DMSO) at a final concentration of 10 mg/mL, diluted in RPMI medium containing 10% fetal calf serum ranging from 100 to 0 µg/mL and incubated with 1 × 10^4^ cells in a 96-well plate. After 18 h of incubation, cell viability was measured using the Cell Proliferation Kit I (MTT) (Sigma, St. Louis, MO, USA), an MTT-based colorimetric assay [36]. Readings were made in a plate reader at 570 nm. All experiments were performed in triplicate. 

## 4. Conclusions

Essential oil from leaves of *C. sylvestris* analyzed in this work showed a different profile in comparison to other previously analyzed oils, being composed by approximately 50% of α-zingiberene. The cytotoxic evaluation displayed that the crude essential oil was active against all cell lines tested, while purified α-zingiberene showed less activity, indicating that there may be synergism of zingiberene with other substances present in the crude oil for activity. For PHZ, the cytotoxic activity decreased, while THZ showed cytotoxic activity against almost all the tested cell lines. These findings suggests that oil from *C. sylvestris* may be an interesting agent for cancer treatment; however additional studies must be done including isolation of active compound, mechanism of action on tumor cells as well as experimental assays.
